# Heterogeneity of γδ T-cell subsets and their clinical correlation in patients with AML

**DOI:** 10.3389/fimmu.2025.1552235

**Published:** 2025-04-01

**Authors:** Siyuan Jiang, Shiyu Zheng, Chao Yao, Dengchong Ning, Shaoyun Zou, Jiannan Zhan, Tianbi Lan, Tingzhuang Yi, Zhenyi Jin, Xiuli Wu

**Affiliations:** ^1^ Institute of Hematology, Medical Laboratory Center, School of Medicine, Jinan University, Guangzhou, China; ^2^ Youjiang Medical University for Nationalities, Baise, China; ^3^ Dongguan Institute of Clinical Cancer Research, Dongguan Key Laboratory of Precision Diagnosis and Treatment for Tumors, The Tenth Affiliated Hospital, Southern Medical University (Dongguan People’s Hospital), Dongguan, China; ^4^ Department of Oncology, Affiliated Hospital of YouJiang Medical University for Nationalities/Key Laboratory of Molecular Pathology in Tumors of Guangxi Higher Education Institutions, Baise, China; ^5^ Department of Pathology, School of Medicine, Jinan University, Guangzhou, China; ^6^ Key Laboratory of Viral Pathogenesis and Infection Prevention and Control (Jinan University), Ministry of Education, Guangzhou, China; ^7^ Jinan-Puhua Joint Laboratory, Guangzhou, China

**Keywords:** acute myeloid leukemia, NKG2D, TIGIT, Foxp3, γδ T cells, prognosis, immune subsets

## Abstract

**Background:**

γδ T cells are integral elements of the immune system and have shown therapeutic potential in the treatment of acute myeloid leukemia (AML). Nevertheless, the influence of distinct functional subsets, including the activating marker NKG2D, the immune exhaustion marker TIGIT, and the regulatory marker Foxp3, on therapeutic outcomes in AML patients remains unknown.

**Methods:**

First, we analyzed RNA-seq data from 167 patients in The Cancer Genome Atlas (TCGA) database, concentrating on the correlations between *NKG2D*, *TIGIT*, and *Foxp3* gene expressions and their association with prognosis in AML. We employed flow cytometry to assess the expression of these molecular markers on γδ T cells and the Vδ1/Vδ2 subsets in the peripheral blood of 25 *de novo* AML (AML-DN) patients, 15 patients in complete remission (CR), and 27 healthy controls (HCs). We also analyzed the relationship between the expression frequencies of NKG2D, TIGIT, and Foxp3 on γδ T cells and their subsets, and their clinical outcomes.

**Results:**

Based on data from TCGA database, we found that a high expression level of NKG2D in combination with a low expression level of TIGIT was significantly associated with longer overall survival (OS) in AML patients. Clinical data revealed that γδ T cells from AML-DN patients exhibited higher expression levels of TIGIT and Foxp3, whereas NKG2D expression was lower compared to that of HCs. Notably, the expression of the NKG2D^+^TIGIT^−^ Vδ1 subset was significantly reduced in AML-DN patients compared to CR patients. Univariate logistic regression and Cox regression analyses further indicated that a high expression of the NKG2D^+^TIGIT^−^ Vδ1 subset was associated with better clinical prognosis.

**Conclusion:**

This study indicates that NKG2D^+^TIGIT^−^ Vδ1 T cells are strongly correlated with improved prognosis in AML, and future research should investigate their potential in adoptive immunotherapy to advance more personalized and precise treatment strategies.

## 1 Introduction

Acute myeloid leukemia (AML) is one of the most prevalent hematological malignancies, characterized by the accumulation of immature myeloid precursors, which leads to the suppression of normal hematopoiesis (1). Despite significant advances in our understanding of AML, the standard treatment regimen remains induction chemotherapy followed by consolidation chemotherapy or hematopoietic stem cell transplantation, with generally poor outcomes, particularly in non-acute promyelocytic leukemia (non-APL; M3) subtypes (2, 3). Additionally, there are currently no effective treatments available to prevent the adverse consequences of relapsed or refractory disease and to achieve sustained complete remission (CR) in AML patients (4). Recent studies have demonstrated that T-cell immunodeficiency is a common feature in AML, leading to the emergence of adoptive T-cell immunotherapy as a promising approach to enhance anti-leukemia therapy (5).

It is well known that γδ T cells are a small subgroup of T cells in human peripheral blood (PB). Human γδ T cells can be divided into two main groups according to their T-cell receptor (TCR) usage of the Vδ1 and Vδ2 chains. The majority of γδ T cells in PB is Vδ2 subset, which is paired with Vγ9 chain. The Vδ1 subgroup exists in the mucosal epithelium and has adaptability (1). As the bridge between adaptive and innate immune systems, activated γδ T cells can promote the anti-tumor function of adaptive immune cells and participate in various immune responses during cancer progression (2). γδ T cells can show broad antigen specificity and natural killer-like (NK-like) cytotoxicity with the absence of the major histocompatibility complex (MHC) molecules, which is why γδ T cells are essential for adoptive T-cell immunotherapy (3, 4). There are many approaches to anti-tumor therapy using γδ T cells, of which the use of chimeric antigen receptor (CAR) T cells has been shown to be well tolerated and highly efficient (3). Despite the advantages that could be exploited, some obstacles need to be addressed for the development of γδ T-cell immunotherapies. One of the most important reasons is that γδ T cells have different functional subsets, and not all the γδ T-cell subsets perform anti-leukemia functions (5). Some expanded γδ T-cell clones and subsets could promote cancer progression by inhibiting anti-tumor responses and enhancing cancer angiogenesis, which may be associated with a poor prognosis of leukemia (6, 7). It needs to be further improved, so it is particularly important to introduce the clustering method of γδ T-cell functional subsets.

Natural killer group 2, member D (NKG2D) is an important activating receptor in natural killer (NK) cells and some kinds of T cells like γδ T cells (8). The engagement of this receptor on NK cells and γδ T cells to legends expressed on tumor cells will induce cell-mediated cytotoxicity and have target cells destroyed (9). In the context of AML, several studies have shown that NKG2D-mediated cytotoxicity of γδ T cells is a key defense mechanism against leukemia (10, 11). T-cell immunoglobulin and immunoreceptor tyrosine-based inhibitory motif domain (TIGIT) is a recently identified immune inhibitory receptor that inhibits immune cell responses at multiple steps of the cancer-immunity cycle (12). TIGIT prevents tumor cell killing by NK cells and cytotoxic T cells and enhances the immune suppressive activity of regulatory T cells through the combination with its legends (13). Our previous research found that TIGIT on memory γδ T_CM_ cells has been associated with poor prognosis in AML (14, 15). High expression of TIGIT on γδ T cells may inhibit their cytotoxic function, leading to impaired immune responses against leukemia cells (16). The regulatory subset of γδ T cells that express the transcription factor forkhead box p3 (Foxp3), termed γδ regulatory T cells (γδ Tregs), has been confirmed to be at low frequencies in tumor-infiltrating leukocytes (TILs) and human PB (17). Similar to the conventional Tregs, inhibitory receptors are expressed on γδ Tregs, and the mechanisms by which their suppressive activity is mediated have been reported (18). Foxp3^+^ Tregs are typically enriched in tumors such as AML and contribute to the formation of an immunosuppressive environment that inhibits anti-tumor immune responses (19). In this study, we hypothetically define the NKG2D^+^ γδ T-cell subset as the activated subset, the TIGIT^+^ γδ T-cell subset as exhausted subsets, and the Foxp3^+^ γδ T-cell subset as the regulatory subset, thus evaluating the expression differences of different functional subsets of γδ T cells in AML patients and healthy controls (HCs) and their correlation with the outcome and prognosis of AML patients. As a result, we can obtain more effective results than traditional clustering and make a more accurate diagnosis and further targeted treatment.

## 2 Materials and methods

### 2.1 Samples

Using RNA-seq data from 167 patients in The Cancer Genome Atlas (TCGA) database, this study analyzed the correlation between the *NKG2D*, *TIGIT*, and *FOXP3* genes, as well as their associations with the prognosis of AML patients. The study included AML patient samples from *de novo* AML (AML-DN) and CR patients, as well as healthy individuals, with participants aged 18 to 90 years. Patients with other underlying conditions or infections were excluded. A total of 25 PB samples were collected from AML-DN patients, including 11 men and 14 women (median age: 61 years, range: 25–88 years) as well as 15 PB samples from CR patients, including eight men and seven women (median age: 54 years, range: 31–79 years) in the First Affiliated Hospital of JNU database from 1 May 2018 to 1 May 2020. The clinical data of the patients are listed in **Table 1**. PB from 27 healthy individuals (HIs) were recruited as controls, with no underlying diseases or infections. The AML patients were diagnosed and classified in accordance with the French–American–British (FAB) classification. There were six pairs of pre- and post-chemotherapy samples in this patient population. The human PB samples were obtained with the consent of both patients and healthy donors. This study was conducted according to the guidelines of the Medical Ethics Committees of the Health Bureau of the Guangdong Province in China, and ethical approval was obtained from the Ethics Committee of the First Affiliated Hospital of Jinan University.

**Table 1:** Clinical information for the AML-DN patients.

No.	Sex	Age	Subtype	WBC (10^9^/L)	BM blast cells (%)	Gene type	Karyotype	Therapy	Disease status	OS (days)
AML1	F	72	M3	5.8	71.5	PML-RARA	t (15,17)	ATRA, ATO	CR	760
AML2	F	62	ND	42.35	27	AML1/ETO	46XY, t (15,18) (q22;23), t (8,21) (q22; q22) [20]	IA	CR	735
AML3	F	25	non-M3	2.79	ND	ND	ND	IA	CR	716
AML4	M	78	M3	5.97	73	PML/RARA	ND	ATRA, ATO	CR	695
AML5	M	36	M3	3.83	21	FLT3-ITDlow, PML/RARA	ND	ATRA, ATO	CR	676
AML6	M	40	M2	4.07	29	JAK/V617f, MLL-ELL	ND	DA	CR	383
AML7	F	37	M5	48.35	63	FLT3-ITD, CEBPA	ND	IA	CR	378
AML8	M	82	M3	32.55	68.5	BCR-ABL, ASXL2, EZH2, TET2	46XY, del (9) (q13)	ATRA, DAC	CR	356
AML9	M	63	M4	40.85	48	FLT3, ITD, NPM1	ND	DCA	CR	216
AML10	F	52	M3	1.13	64	NRAS, TP53, STAG2	ND	IA	CR	186
AML11	M	43	M2	3.67	ND	K-RAS, DEK-CAN, E2A/HLF	ND	HSCT	CR	389
AML12	F	44	M1	34.22	95	FLT3-ITDlow, CEBPA-N	ND	DHA	Non-CR	456
AML13	F	47	M3	ND	ND	ND	ND	ND	Non-CR	10
AML14	M	65	ND	2.34	78.5	NPM1, IDHI, DNMT3A	Abnormal	DA	Non-CR	709
AML15	F	56	ND	1.02	23.5	TET2, SF3B1	ND	DCGA	Non-CR	120
AML16	F	33	M2b	20.8	27.5	AML1-ETO	ND	Untreated	Non-CR	90
AML17	F	67	M5	76.14	97.5	MLL/AF9, EVI1	ND	Ara-C	Non-CR	6
AML18	M	77	M5	36.48	ND	ND	47, XY, +8(10)	IA	Non-CR	196
AML19	F	61	ND	2.8	22	TP53	5q-	DCAG	Non-CR	146
AML20	M	70	M2	25.85	92.5	MLL/AF10, WT1	44, X, −Y, add (10) (p14), −11[4]/45, idem, +mar [6]	Ara-C	Non-CR	21
AML21	M	67	M5	45.63	ND	ND	ND	Conservative treatment	Non-CR	116
AML22	M	64	M4	6.43	34	ND	ND	IA	Non-CR	35
AML23	F	47	M2	5.68	ND	ND	46, XX (3)	DA	Non-CR	160
AML24	F	88	M5	45.94	62.5	ND	ND	Conservative treatment	Non-CR	13
AML25	F	43	ND	28.89	5	AML1/ETO, TET2, RUNX1/RUNX1T1	ND	Chemotherapy	Non-CR	158

WBC, white blood cell; BM, bone marrow; F, female; M, male; ND, not detected; HSCT, hematopoietic stem cell transplantation; AML, acute myeloid leukemia; M0, minimally differentiated AML; M1, AML without maturation; M2, AML with maturation; M3, acute promyelocytic leukemia; M4, acute myelomonocytic leukemia; M5, acute monocytic leukemia; CR, complete remission; non-CR, non-complete remission;/, unknown; ATRA, all-*trans*-retinoic acid; ATO, arsenic trioxide; DA, daunorubicin + cytarabine; DCAG, decitabine + aclarubicin + cytarabine + recombinant granulocyte colony-stimulating factor; IA, idarubicin + cytarabine; DCA, decitabine; Ara-C, cytarabine; DAC, decitabine.

### 2.2 Flow cytometry analysis

Peripheral blood mononuclear cells (PBMCs) were isolated from AML patients and HIs and then incubated with the following antibodies: CD3-APC-H7 (Clone SK7), TCR γδ-PerCP-Cy5.5 (Clone B1), Vδ1-FITC (Clone TS8.2), Vδ2-PE-Cy7 (Clone B6), NKG2D-BV510 (Clone 1D11), TIGIT-BV421 (Clone A15153G), BV510 isotype control (Clone MOPC-21), and BV421 isotype control (Clone G155-178) (BioLegend, San Diego, CA, USA; BD Biosciences, San Jose, CA, USA). Extracellular staining was carried out according to the instructions of the manufacturers. Five microliters of each conjugated fluorescent antibody mentioned above were incubated with 300 µL of each PB sample at room temperature for 20 minutes in the dark. Three milliliters of 1 × RBC Lysis Buffer (BD Biosciences, USA) was used for lysing erythrocytes for 10 minutes in the dark. Samples were completely washed with PBS, followed by centrifugation at 350 × *g* for 5 minutes. The cells were first surface stained as described above. Then, 500 µL of Fixation Buffer (BioLegend, USA) was added, and the cells were incubated in the dark for 20 minutes. Following fixation, 1 mL of Intracellular Staining Perm Wash Buffer (1×) (BioLegend, USA) was added, and the cells were centrifuged at 350 × *g* for 5 minutes. The supernatant was discarded, and 5 µL of Foxp3-AF647 (Clone 150D) (BioLegend, San Diego, CA, USA) flow cytometry antibody was added to the cells. In the isotype control tube, 5 µL of AF647 isotype control (Clone MOPC-21) (BioLegend, San Diego, CA, USA) was added. The samples were mixed thoroughly and incubated at room temperature, protected from light, for 20 minutes. After incubation, the cells were washed twice with 1 mL of Intracellular Staining Perm Wash Buffer (1×) by centrifuging at 350 × *g* for 5 minutes each time. Then, all samples were resuspended with 200 µL PBS for analysis by flow cytometry. All samples were analyzed using a BD Verse flow cytometer (BD Biosciences, USA), and the FlowJo version 10.8.1. software (Treestar) was used to analyze the data.

### 2.3 Bioinformatics analysis

The clinical data and RNA expression profiles of 167 AML patients were obtained from TCGA database. The relationships among the *NKG2D*, *TIGIT*, and *FOXP3* genes, as well as their associations with the prognosis of AML patients, were assessed using the Kaplan–Meier survival curve analysis and Pearson’s correlation analysis.

### 2.4 Statistical analysis

All data are represented as medians. Groups were tested for normal distribution using the Kolmogorov–Smirnov test. Statistical differences between two and among three groups were analyzed using the Mann–Whitney U and Kruskal–Wallis tests, respectively. Appropriate corrections for multiple comparisons were also made using Dunn’s multiple comparisons test method. For paired samples, the Wilcoxon signed-rank test was used for comparison. Spearman’s rank coefficient was used to analyze correlations. Logistic regression was used to analyze the associations between the frequencies of γδ T cells and their subsets and the outcome of AML patients performed. The Cox regression method was used to analyze between-group survival differences. The explanatory variables included the proportions of γδ T cells and their subgroups. For the AML RNA-seq data in TCGA database, the optimal cutoff value was determined based on the minimum *p*-value obtained from the Cox proportional hazards survival analysis. Subsequently, the samples were categorized into high- and low-expression groups according to this optimal threshold. Then, the relationship between the expression levels of the *NKG2D*, *TIGIT*, and *FOXP3* genes and the clinical prognosis of AML patients was determined using the Kaplan–Meier survival analysis, which was performed using the R package survminer. The correlation among these three genes was also analyzed using Pearson’s correlation analysis. All statistical tests were two-tailed. *p*-Values less than 0.05 were considered statistically significant (*p < 0.05; **p < 0.01; ***p < 0.001). All calculations were performed using the GraphPad Prism 10.3 software (GraphPad Software Inc., San Diego, CA, USA) and SPSS.

## 3 Results

### 3.1 Co-expression characteristics of *NKG2D*, *TIGIT*, and *FOXP3* genes in AML

**Figure 1:**
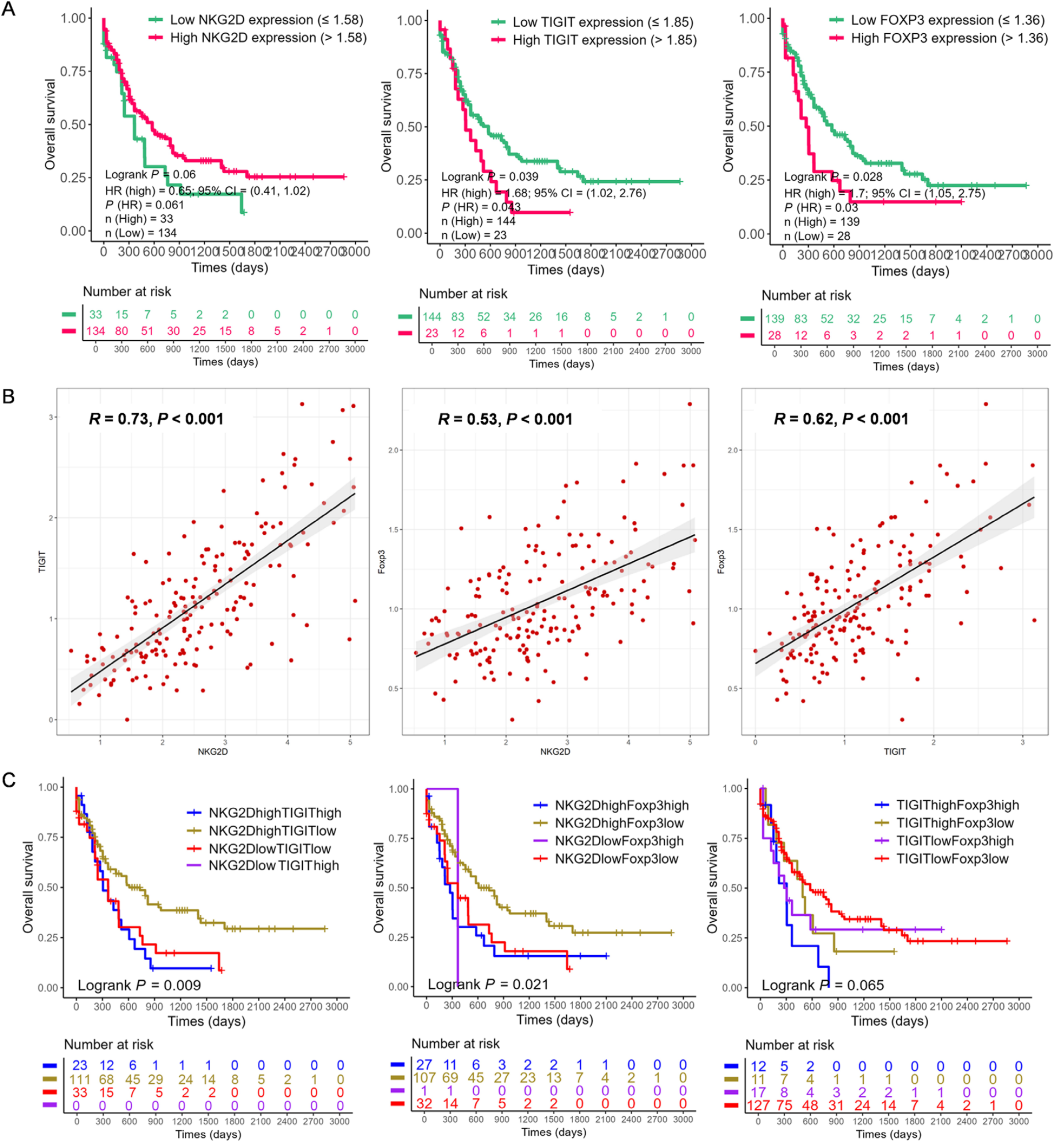
The correlation and prognosis of *NKG2D*, *TIGIT*, and *FOXP3* gene expression in AML based on The Cancer Genome Atlas (TCGA) data. **(A)** Based on the optimal cut-off value of the gene expression levels, the *NKG2D*, *TIGIT*, and *FOXP3* genes were classified into high expression (red line) and low expression (green line) groups, which were plotted in Kaplan–Meier curves (top) with the number of AML patients at risk (bottom). For *NKG2D*, high *vs.* low expression showed a trend toward better prognosis (*p* = 0.06). For TIGIT and FOXP3, high expression was significantly associated with prognosis (*p* = 0.039 and *p* = 0.028, respectively). **(B)** Correlation analysis of the expression levels of *NKG2D*, *TIGIT*, and *FOXP3* genes. **(C)** Quartile analysis of gene pairs *(NKG2D/TIGIT*, *NKG2D/FOXP3*, and *TIGIT/FOXP3*) was conducted to assess the impact on AML prognosis. The gene pairs were categorized into four groups based on high or low expression levels determined by the best cut-off value. Among these, the *NKG2D* ^high^/*TIGIT* ^low^ group (*p* = 0.009) and the *NKG2D* ^high^/*FOXP3* ^low^ group (*p* = 0.021) exhibited significantly better prognoses. AML, acute myeloid leukemia.

RNA-seq data were first utilized from 167 AML patients in TCGA-LAML database (https://portal.gdc.cancer.gov/) to investigate the relationship between *NKG2D*, *TIGIT*, and *FOXP3* gene expression levels and their prognostic implications. Based on the optimal cut-off values for the expression levels of these genes, patients were categorized into high- and low-expression groups. Subsequently, the Kaplan–Meier survival curves were constructed for comparative analysis. Initial analysis revealed a positive correlation between elevated *NKG2D* expression and improved overall survival (OS) (*NKG2D* ^high^ *vs. NKG2D* ^low^, 2-year OS: 43.3% *vs.* 25.9%, *p* = 0.060) (**Figure 1A**). Conversely, increased levels of *TIGIT* (*TIGIT* ^high^ *vs. TIGIT* ^low^, 2-year OS: 19.4% *vs.* 43.6%, *p* = 0.039) and *FOXP3* (*FOXP3* ^high^ *vs. FOXP3* ^low^, 2-year OS: 19.8% *vs.* 43.9%, *p* = 0.028) (**Figure 1A**) were associated with poorer OS. Significant positive correlations were observed between *NKG2D* and *TIGIT* (*R* = 0.73, *p* < 0.001) (**Figure 1B**), *NKG2D* and *FOXP3* (*R* = 0.53, *p* < 0.001), and *TIGIT* and *FOXP3* (*R* = 0.62, *p* < 0.001) (**Figure 1B**).

Further analysis categorized patients into groups based on the single high expression, single low expression, co-high expression, and co-low expression of *NKG2D*, *TIGIT*, and *FOXP3* genes. The Kaplan–Meier analysis indicated that *NKG2D* ^high^ *TIGIT* ^low^ expression was associated with better OS (*p* = 0.009) (**Figure 1C**), as well as *NKG2D* ^high^ *FOXP3* ^low^ expression (*p* = 0.021), while *NKG2D* ^low^ *FOXP3* ^high^ expression correlated significantly with poorer OS (*p* = 0.021) (**Figure 1C**). Additionally, patients with higher levels of *TIGIT* ^low^ *FOXP3* ^low^ genes showed better OS (*p* = 0.065) (**Figure 1C**), whereas those with higher levels of *TIGIT* ^high^ *FOXP3* ^high^ genes had poorer OS (*p* = 0.065) (**Figure 1C**). These findings underscore the potential prognostic significance of *NKG2D*, *TIGIT*, and *FOXP3* gene expression levels in AML, suggesting their roles in disease progression and clinical outcomes.

### 3.2 Distribution pattern of γδ T-cell subsets in AML

**Figure 2:**
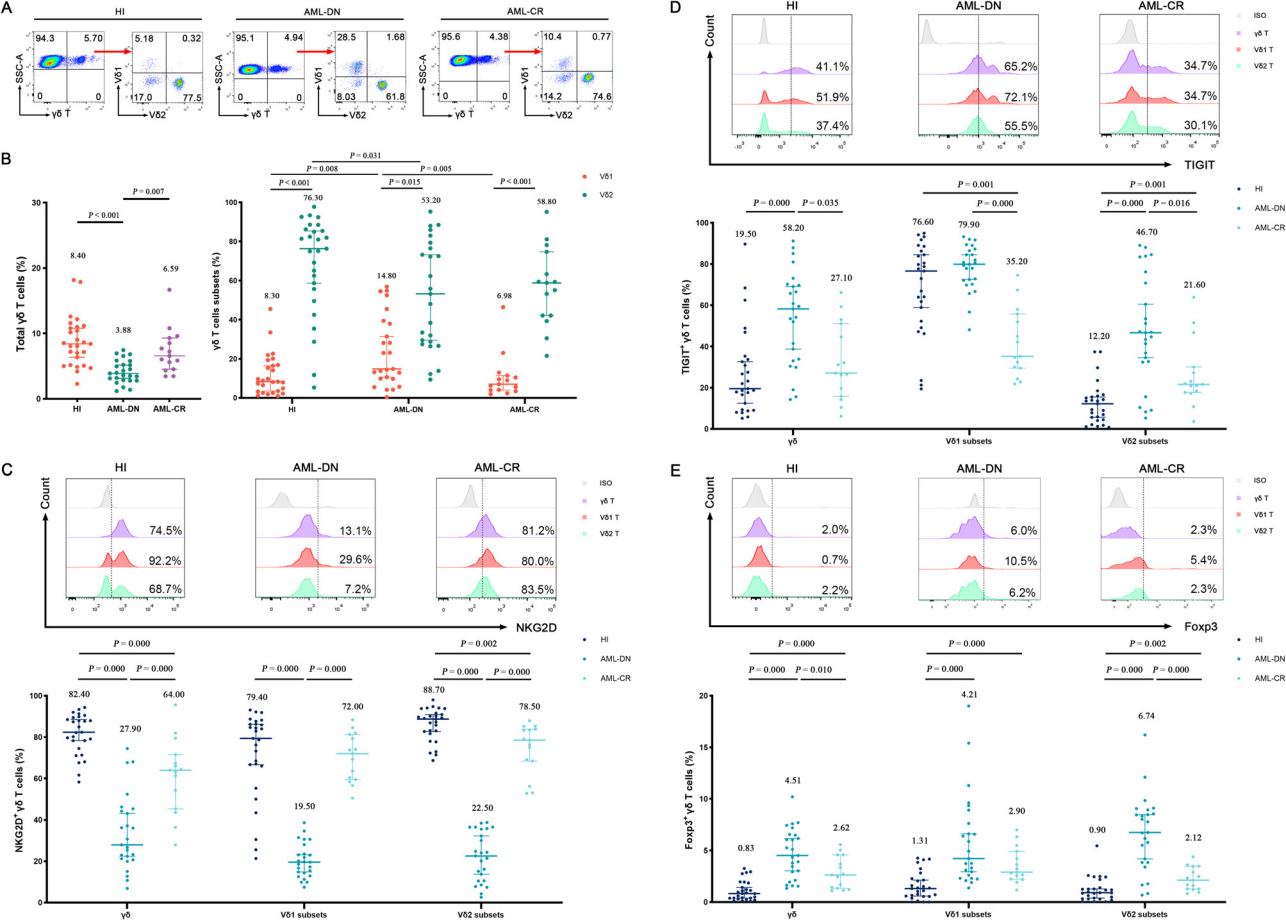
Distribution and frequency of NKG2D, TIGIT, and Foxp3 expression in γδ T-cell, Vδ1, and Vδ2 cell subpopulations from PB of AML-DN patients, CR patients, and HIs. **(A)** γδ T cells were gated from CD3^+^ T cells and further identified as Vδ1 and Vδ2 subpopulations in HIs and AML patients using flow cytometry analysis. **(B)** Comparison of the percentages of γδ T cells and their subsets (Vδ1 and Vδ2), both of which are derived from the total γδ T cells on the left, across AML-DN patients, CR patients, and HIs. **(C–E)** Expression levels of NKG2D, TIGIT, and FOXP3 in γδ T cells and their subsets (Vδ1 and Vδ2) were analyzed for AML-DN patients, CR patients, and HIs. The numbers above the scatter plots represent the median value of the data for each group. Statistical analyses were performed using the unpaired Mann–Whitney U test **(B–E)**. PB, peripheral blood; AML-DN, *de novo* acute myeloid leukemia; CR, complete remission; HIs, healthy individuals.

In this study, we characterized the distributions of γδ T and their cell subsets in PB from HIs (n = 27), untreated AML-DN patients (n = 25), and CR patients (n = 15), which included six paired samples of AML-DN and CR. A decreased percentage of γδ T cells was found in the AML-DN group compared to HIs (median: 3.88% *vs*. 8.40%, *p* < 0.001) and AML-CR (median: 3.88% *vs.* 6.59%, *p* = 0.007) (**Figures 2A, B**). In addition, the proportion of Vδ1 cells was higher in the AML-DN group when compared with HIs (median: 14.80% *vs.* 8.30%, *p* = 0.008) and the CR group (median: 14.80% *vs*. 6.98%, *p* = 0.005). A lower frequency of Vδ2 cells was found in the AML-DN group compared with the HIs (median: 53.20% *vs*. 76.30%, *p* = 0.031) (**Figure 2B**). We further analyzed the expression of the non-Vδ1/Vδ2 subset and found no significant differences among the AML-DN, CR, and HI groups (**Supplementary Figure S1A**).

We further accessed NKG2D, TIGIT, and Foxp3 expression patterns on γδ T cells in the AML group and HIs. First, the similar expression pattern of NKG2D on γδ T and its subset cells was AML-DN (median: 27.90%) < AML-CR (median: 64.00%) < HIs (median: 82.40%). Lower frequencies of NKG2D^+^ γδ (median: 27.90%), NKG2D^+^ Vδ1 (median: 19.50%), and NKG2D^+^ Vδ2 (median: 22.50%) in the AML-DN group were found compared with those in HIs and the CR group (**Figure 2C**; **Supplementary Figure S2**). Furthermore, the frequency of NKG2D^+^ Vδ2 in CR was statistically decreased compared with that of HIs (median: 78.50% *vs.* 88.70%, *p* = 0.002) (**Figure 2C**). Second, the expression patterns of TIGIT on γδ T and its subset cells were different from NKG2D expression, which showed HIs (median: 19.50%) < AML-CR (median: 27.1%) < AML-DN group (median: 58.20%) (**Figure 2D**; **Supplementary Figure S2**). A higher tendency of TIGIT^+^ γδ (median: 58.20% *vs.* 19.50%, *p* = 0.000), TIGIT^+^ Vδ1 (median: 79.90% *vs.* 76.60%, *p* = 0.193), and TIGIT^+^ Vδ2 (median: 46.70% *vs.* 12.20%, *p* = 0.000) was found in the AML-DN group compared with HIs (**Figure 2D**). Similar expression patterns of Foxp3 on γδ T and its subset cells were also found in AML, which showed HIs (median: 0.83%) < AML-CR (median: 2.62%) < AML-DN (median: 4.51%) (**Figure 2E**; **Supplementary Figure S2**). In the AML-DN group, there was a dramatically increased trend in Foxp3^+^ Vδ1 in the γδ T-cell subset (median: 4.21% *vs.* 1.31%, *p* = 0.000) and Foxp3^+^ Vδ2 in the γδ T-cell subset (median: 6.74% *vs.* 0.90%, *p* = 0.000) (**Figure 2E**). In the CR group, the Foxp3^+^ population was also found to be higher than these three subgroups in γδ T cells (median: 2.90% *vs*. 1.31%, *p* = 0.000; median: 2.12% *vs*. 0.90%, *p* = 0.002) (**Figure 2E**).

Interestingly, we found that the expression patterns of NKG2D, TIGIT, and Foxp3 in the non-Vδ1/Vδ2 subset were similar to those in the Vδ1 and Vδ2 subsets. Compared to the HIs, the percentage of NKG2D^+^ non-Vδ1/Vδ2 T cells was significantly decreased in the AML-DN group (median: 27.50% *vs.* 72.00%, *p* < 0.001), while the percentages of TIGIT^+^ non-Vδ1/Vδ2 T cells (median: 53.00% *vs.* 35.40%, *p* < 0.001) and Foxp3^+^ non-Vδ1/Vδ2 T cells (median: 5.87% *vs.* 0.99%, *p* < 0.001) were significantly increased. The expression trends in the CR group were intermediate between those of AML-DN and HIs (**Supplementary Figure S1B**). These findings revealed the distribution patterns of γδ T cells and their subpopulations in different disease states and explored the differences in the expression of marker molecules such as NKG2D, TIGIT, and Foxp3.

### 3.3 Relevance of NKG2D and TIGIT/Foxp3 co-expression on γδ T-cell subsets

**Figure 3:**
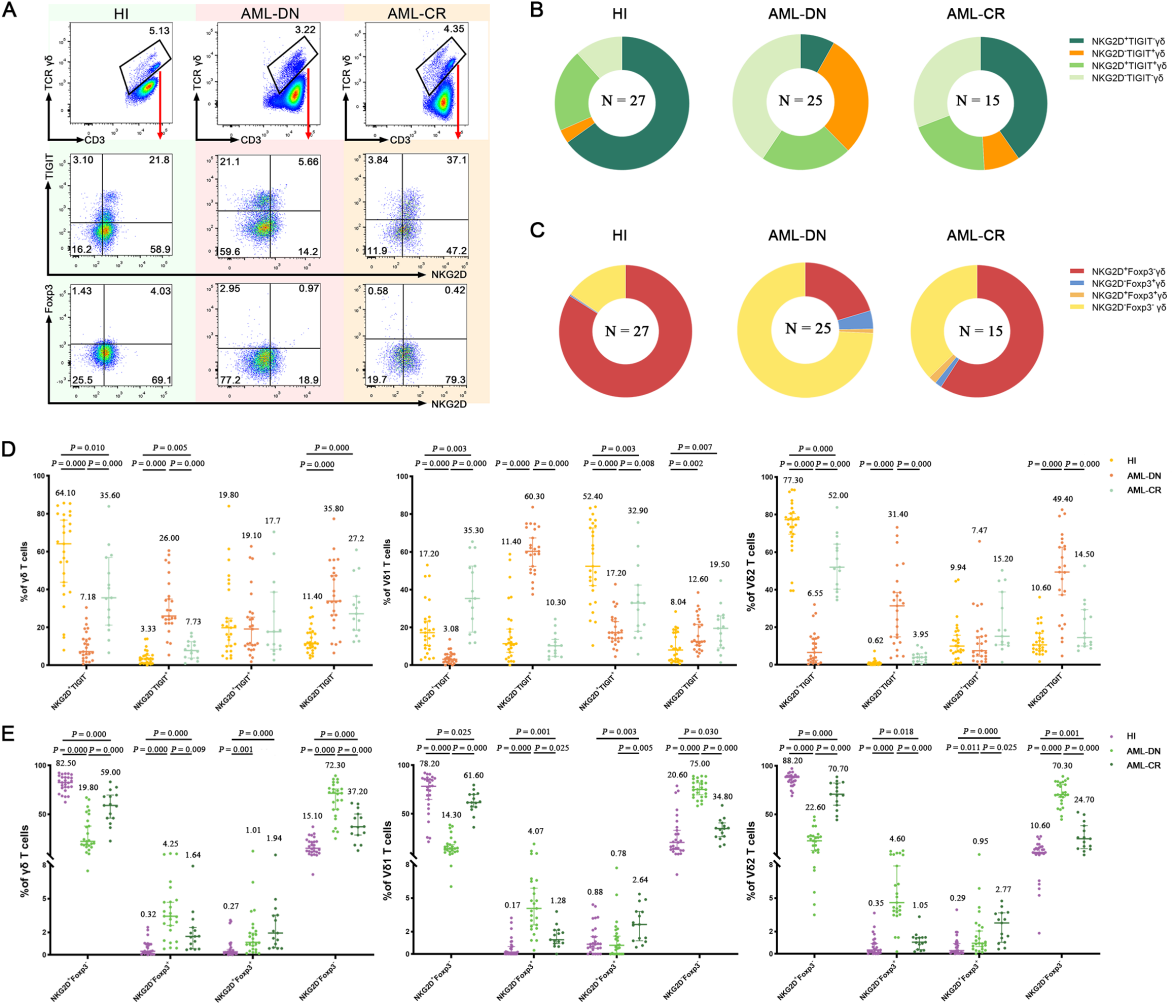
Co-expression of NKG2D, TIGIT, and Foxp3 in γδ, Vδ1, and Vδ2 cell subsets among AML-DN patients, CR patients, and HIs. **(A)** Flow cytometry analysis displaying the co-expression of NKG2D, TIGIT, and Foxp3 in γδ, Vδ1, and Vδ2 cell subsets from AML-DN patients, CR patients, and HIs. In this study, the gating strategy was carefully designed to ensure accurate identification of γδ T-cell subsets and their relevant markers. The gating was performed as follows: we first gated on CD3^+^ cells and further selected for γδ T cells by analyzing the CD3^+^/γδ T-cell population. Within the γδ T-cell population, Vδ1 and Vδ2 cells were identified based on specific marker expression, allowing for the analysis of these subsets individually. Subsequent gates were applied to identify the expression of NKG2D, TIGIT, and Foxp3 within the total γδ T-cell population, as well as separately within the Vδ1 and Vδ2 subsets. The specificity of antibody staining was validated using isotype-matched controls, as demonstrated in **Supplementary Figure S1**. **(B)** Pie charts illustrating the proportion of NKG2D, TIGIT, and Foxp3 co-expression in γδ, Vδ1, and Vδ2 subsets in AML-DN patients, CR patients, and HIs. **(C)** Quantitative comparison of NKG2D, TIGIT, and Foxp3 co-expression in total γδ, Vδ1, and Vδ2 subsets among AML-DN patients, CR patients, and HIs. The numbers above the scatter plots represent the median value of the data for each group **(D, E)**. Data are presented as medians. The data were analyzed using the unpaired Mann–Whitney U test **(B–E)**. AML-DN, *de novo* acute myeloid leukemia; CR, complete remission; HIs, healthy individuals.

To further assess whether the co-expression of NKG2D and TIGIT on the surface of γδ T cells may correlate with the prognosis of AML, we classified γδ T cells into four distinct subsets: NKG2D^−^TIGIT^+^, NKG2D^+^TIGIT^+^, NKG2D^+^TIGIT^−^, and NKG2D^−^TIGIT^−^ (**Figures 3A, B, D**; **Supplementary Figure S2**). There, we especially focused on two simple positive subsets: NKG2D^+^TIGIT^−^ and NKG2D^−^TIGIT^+^. Interestingly, we observed a significant decrease in the frequency of NKG2D^+^TIGIT^−^ subsets within the total γδ T cells (median: 7.18% *vs.* 64.10%, *p* = 0.000), as well as in the Vδ1 T cells (median: 3.08% *vs.* 17.20%, *p* = 0.000) and Vδ2 T cells (median: 6.55% *vs.* 77.30%, *p* = 0.000) in the AML-DN group compared to HIs (**Figure 3D**). In contrast, the frequency of NKG2D^−^TIGIT^+^ subsets was significantly higher in the total γδ T cells (median: 26.00% *vs.* 3.33%, *p* = 0.000), as well as in the Vδ1 T cells (median: 60.30% *vs.* 11.40%, *p* = 0.000) and Vδ2 T cells (median: 31.40% *vs.* 0.62%, *p* = 0.000) in the AML-DN group compared to HIs (**Figure 3D**). Compared with the CR group, the tendency of NKG2D^+^TIGIT^−^ subsets expressing on the surface of total γδ T cells (median: 7.18% *vs.* 35.60%, *p* = 0.000), Vδ1 T cells (median: 3.08% *vs*. 35.30%, *p* = 0.000), and Vδ2 T cells (median: 6.55% *vs.* 52.00%, *p* = 0.000) was lower in the AML-DN group (**Figure 3D**). Likewise, the frequencies of NKG2D^−^TIGIT^+^ subsets expressing on the surface of total γδ T cells (median: 26.0% *vs.* 7.73%, *p* = 0.000), Vδ1 T cells (median: 60.30% *vs*. 10.30%, *p* = 0.000), and Vδ2 T cells (median: 31.40% *vs.* 3.95%, *p* = 0.000) were higher in the AML-DN group compared with the CR group (**Figure 3D**). We also observed the same pattern in the non-Vδ1/Vδ2 subset. Compared to AML-DN group, the percentage of NKG2D^+^TIGIT^−^ non-Vδ1/Vδ2 T-cell subset was significantly increased in HIs (median: 23.90% *vs.* 7.50%, *p* < 0.001), whereas the proportion of NKG2D^−^TIGIT^+^ non-Vδ1/Vδ2 T-cell subset was significantly decreased (median: 31.10% *vs.* 5.53%, *p* < 0.001) (**Supplementary Figure S1C**).

Similarly, we described the expression difference of the other two single-positive subsets: NKG2D^+^Foxp3^−^ and NKG2D^−^Foxp3^+^ in different γδ T-cell subgroups among His and the AML-DN and CR groups (**Figures 3A, C, E**; **Supplementary Figure S2**). We discovered that NKG2D^+^Foxp3^−^ decreased in all γδ T subgroups: total γδ T cells (median: 19.80% *vs.* 82.50%, *p* = 0.000), Vδ1 T cells (median: 14.30% *vs*. 78.20%, *p* = 0.000), and Vδ2 T cells (median: 22.60% *vs.* 88.20%, *p* = 0.000) in the AML-DN group compared with the HIs (**Figure 3E**). Inversely, the frequency of NKG2D^−^Foxp3^+^ was increased in total γδ T cells (median: 4.25% *vs.* 0.32%, *p* = 0.000), Vδ1 T cells (median: 4.07% *vs.* 0.17%, *p* = 0.000), and Vδ2 T cells (median: 4.60% *vs.* 0.35%, *p* = 0.000) in the AML-DN group compared with the HIs (**Figure 3E**). Comparably, the lower tendency of NKG2D^+^Foxp3^−^ subsets has been showed in total γδ T cells (median: 19.80% *vs.* 59.00%, *p* = 0.000), Vδ1 T cells (median: 14.30% *vs*. 61.60%, *p* = 0.000), and Vδ2 T cells (median: 22.60% *vs.* 75.00%, *p* = 0.000) in the AML-DN group compared with the CR group (**Figure 3E**). Simultaneously, a higher tendency of NKG2D^−^Foxp3^+^ subsets has been shown in total γδ T cells (median: 4.25% *vs.* 1.64%, *p* = 0.009), Vδ1 T cells (median: 4.07% *vs.* 1.28%, *p* = 0.025), and Vδ2 T cells (median: 4.60% *vs.* 1.05%, *p* = 0.000) in the AML-DN group compared with the CR group (**Figure 3E**). We further analyzed the correlation between the co-expression of NKG2D and Foxp3 in the non-Vδ1/Vδ2 subset. The results showed that the percentage of NKG2D^+^Foxp3^−^ non-Vδ1/Vδ2 T cells was significantly reduced in both the AML-DN group (median: 26.80% *vs.* 71.30%, *p* < 0.001) and the CR group (median: 39.80% *vs.* 71.30%, *p* = 0.001) compared to HIs. In contrast, the percentage of NKG2D^−^Foxp3^+^ non-Vδ1/Vδ2 T cells was significantly increased in both the AML-DN group (median: 4.90% *vs.* 0.46%, *p* < 0.001) and the CR group (median: 2.17% *vs.* 0.46%, *p* = 0.001) compared to HIs (**Supplementary Figure S1D**).

### 3.4 Influence of TIGIT, Foxp3, and NKG2D on γδ T-cell subsets in AML patients before and after treatment and clinical outcomes

**Figure 4:**
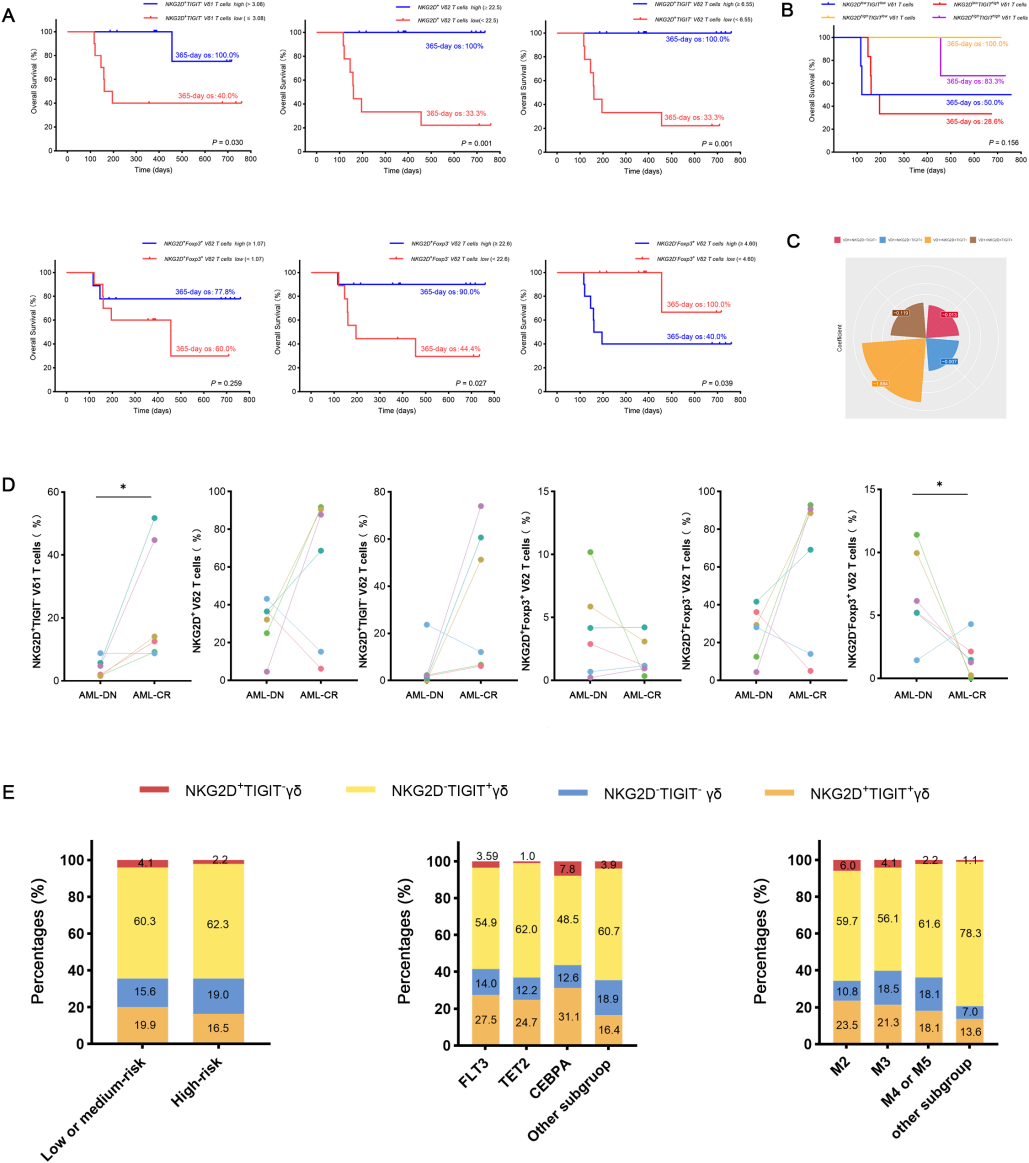
Analysis of OS and clinical outcomes in subgroups of AML-DN patients, focusing on NKG2D and TIGIT co-expression in Vδ1 T cells. **(A)** Kaplan–Meier analysis of OS stratified by the high and low expression of NKG2D^+^TIGIT^−^ Vδ1 T cell subgroups (NKG2D^+^TIGIT^−^ Vδ1 T^high^ *vs.* NKG2D^+^TIGIT^−^ Vδ1 T^low^) based on the median value. A significant difference in 365-day OS was observed (100% *vs.* 40%, *p* = 0.03). **(B)** Correlation between the proportions of the four subgroups defined by NKG2D and TIGIT co-expression in Vδ1 T cells and OS in AML-DN patients. **(C)** Contribution coefficient analysis of the NKG2D^+^ and TIGIT^−^ co-expressing subpopulation within Vδ1 T cells to the prognosis of AML. **(D)** Paired analysis of clinical outcomes and prognoses in AML patients before and after treatment, highlighting subgroup dynamics. **(E)** Subgroup analysis of gene mutations, FAB classification, and risk stratification in AML patients, focusing on the NKG2D/TIGIT combined expression subgroups. OS, overall survival; AML-DN, *de novo* acute myeloid leukemia. *p < 0.05.

Despite the increased insight into the phenotype of γδ T cells, whether these phenotypes correlate with clinical outcomes remains poorly understood. Therefore, we assessed the clinical outcomes of the 25 AML-DN patients (**Table 1**) and further analyzed the association among AML outcome, OS, and different subsets of γδ T cells. Logistic regression analyses indicated that several factors are associated with the clinical outcomes and prognosis of AML (**Table 2**). We further investigated whether the γδ T cells could be associated with OS. We selected 19 patients with OS exceeding 100 days from 25 AML-DN patients and then divided the 19 AML-DN patients into high and low groups based on the median frequency of the γδ T cells. There was a significant difference in the NKG2D^+^ γδ T-cell group (NKG2D^+high^ γδ T cell *vs.* NKG2D^+low^ γδ T cell 365-day OS: 100.0% *vs.* 29.6%, *p* = 0.003), NKG2D^+^ Vδ1 T cells (NKG2D^+high^ Vδ1 T cell *vs.* NKG2D^+low^ Vδ1 T cell 365-day OS: 100.0% *vs*. 37.5%, *p* = 0.017), and NKG2D^+^ Vδ2 T cells (NKG2D^+high^ Vδ2 T cell *vs.* NKG2D^+low^ Vδ2 T cell 365-day OS: 100.0% *vs.* 33.3%, *p* = 0.001) (**Figure 4A**). Furthermore, we analyzed the expression of NKG2D, TIGIT, and Foxp3 and found that the pairwise combinations of these three immune checkpoints connected with OS in AML patients.

**Table 2:** Binary logistic regression analysis of the relationship between γδ T-cell subsets and clinical efficacy in AML-DN patients.

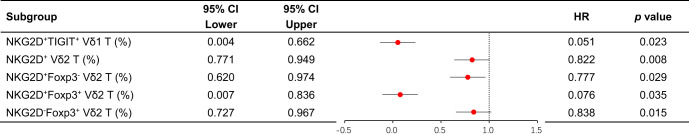

AML, acute myeloid leukemia.

Among patients with low expression of NKG2D in total γδ T cells and Vδ2 T cells, concomitant high expression of TIGIT correlated with poor OS (NKG2D^high^TIGIT^low^ γδ T cell *vs.* NKG2D^low^TIGIT^high^ γδ T cell 365-day OS: 100.0% *vs.* 50.0%, *p* = 0.003; NKG2D^high^TIGIT^low^ Vδ2 T cell *vs.* NKG2D^low^TIGIT^high^ Vδ2 T cell 365-day OS: 100.0% *vs.* 50.0%, *p* = 0.032) (**Figure 4B**). Patients with low expression of NKG2D in Vδ2 T cells, together with high expression of Foxp3, also related to poor OS (NKG2D^high^Foxp3^low^ Vδ2 T cell *vs*. NKG2D^low^Foxp3^high^ Vδ2 T cell 365-day OS: 100.0% *vs.* 50.0%, *p* = 0.032) (**Figure 4B**). Moreover, univariate Cox regression analysis indicated that the frequencies of NKG2D^+^ γδ T cells, NKG2D^+^ Vδ2 T cells, NKG2D^+^TIGIT^−^ Vδ1 T cells, NKG2D^−^Foxp3^+^ Vδ2 T cells, and NKG2D^+^Foxp3^−^ Vδ2 T cells had significant differences in survival rates and were independent prognostic risk factors for AML prognosis (**Table 3**). A notable discrepancy was found among the NKG2D^−^TIGIT^+^ γδ T cells (NKG2D^−^TIGIT^+high^ γδ T cell *vs.* NKG2D^−^TIGIT^+low^ γδ T cell 365-day OS: 40.0% *vs*. 100.0%, *p* = 0.003) (**Figure 4A**). NKG2D^+^TIGIT^−^ γδ T cells (NKG2D^+^TIGIT^−high^ γδ T cell *vs.* NKG2D^+^TIGIT^−low^ γδ T cell 365-day OS: 90.0% *vs.* 44.4%, *p* = 0.035), NKG2D^+^TIGIT^−^ Vδ1 T cells (NKG2D^+^TIGIT^−high^ Vδ1 T cell *vs*. NKG2D^+^TIGIT^−low^ Vδ1 T cell 365-day OS: 100.0% *vs.* 40.0%, *p* = 0.030), and NKG2D^+^TIGIT^−^ Vδ2 T cells (NKG2D^+^TIGIT^−high^ γδ T cell *vs.* NKG2D^+^TIGIT^−low^ γδ T cell 365-day OS: 100.0% *vs.* 33.3%, *p* = 0.001) also exhibited significant differences (**Figure 4A**). Also, the expression levels of NKG2D^+^Foxp3^−^ Vδ2 T cells (NKG2D^+^Foxp3^−high^ Vδ2 T cell *vs.* NKG2D^+^Foxp3^−low^ Vδ2 T cell 365-day OS: 90.0% *vs.* 44.4%, *p* = 0.027) and NKG2D^−^Foxp3^+^ Vδ2 T cells (NKG2D^−^Foxp3^+high^ T cell *vs.* NKG2D^−^Foxp3^+low^ T cell 365-day OS: 40.0% *vs.* 100.0%, *p* = 0.039) correlate significantly with OS (**Figure 4A**).

**Table 3:** Univariate Cox proportional hazards regression analysis of clinical treatment outcomes in AML-DN patients.

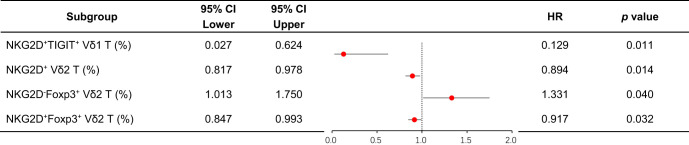

AML-DN, de novo acute myeloid leukemia.

To further confirm the relevance of γδ T-cell subsets and clinical outcomes of AML patients, and meanwhile determine changes in the γδ T-cell subsets by chemotherapy, paired comparisons of the percentage of different γδ T-cell populations were conducted in six patients before and after chemotherapy. Notably, there was an increase in the frequencies of NKG2D^+^TIGIT^−^ Vδ1 cells and NKG2D^+^Foxp3^−^ Vδ2 cells in AML patients who achieved CR after chemotherapy (n = 6), which informed that these two subsets were intimately correlated with better outcomes (**Figure 4D**). The results suggest that the NKG2D^+^TIGIT^−^ Vδ1 T-cell subset is a relatively sensitive survival predictor in AML. Subsequently, we compared the contribution coefficient analysis of the NKG2D/TIGIT co-expressing subpopulation in Vδ1 and found that the contribution coefficient of the NKG2D^+^TIGIT^−^ Vδ1 cell subset was the highest, with the best prognosis in AML patients (**Figure 4C**). Therefore, we determined whether the subpopulations co-expressing NKG2D and TIGIT in Vδ1 exhibited different clinical characteristics. The results showed that the proportion of NKG2D^+^TIGIT^−^ Vδ1 subpopulation was elevated in the low- or intermediate-risk groups. Compared to the TET and FLT3 genotypes, the proportion of NKG2D^+^TIGIT^−^ Vδ1 subpopulation was higher in the CEBPA genotype group. In the FAB classification, the proportion was significantly lower in the M4 or M5 subgroups compared to the M2 and M3 subgroups (**Figure 4E**).

## 4 Discussion

**Figure 5:**
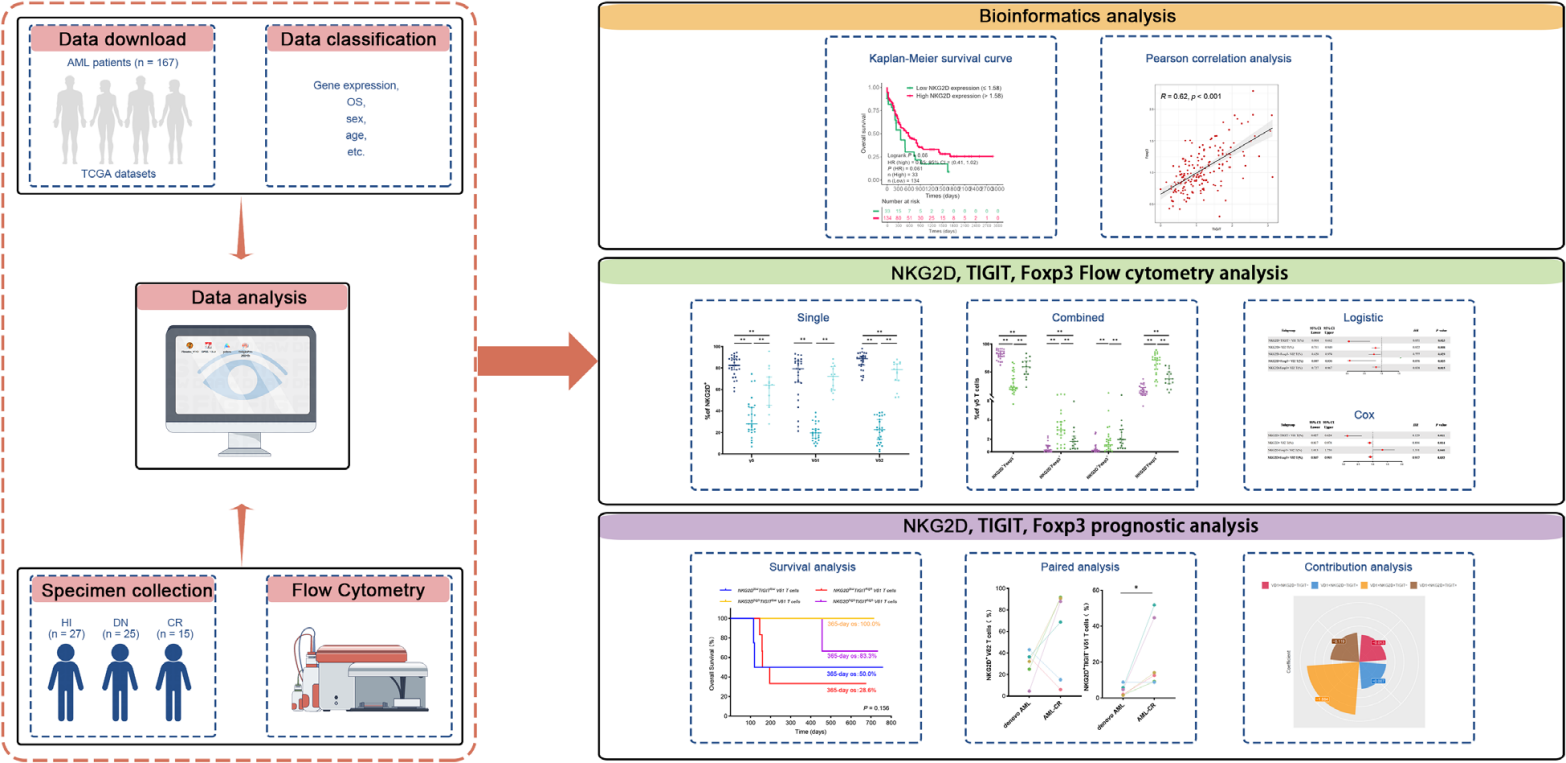
This study design schematic outlines the methodology. RNA-seq data from AML patients were downloaded from TCGA database, and clinical information was integrated to investigate the correlation between the expression of *NKG2D*, *TIGIT*, and *FOXP3* genes and their relationship with clinical prognosis in AML patients. Flow cytometry analysis was conducted to evaluate the expression of molecular markers (NKG2D, TIGIT, and FOXP3) on γδ T cells and their Vδ1 and Vδ2 subpopulations in PB samples collected from 25 AML-DN patients, 15 CR patients, and 27 HIs. The analysis included both individual markers and their paired combinations. Paired samples from six patients who achieved CR after treatment were further analyzed to explore correlations with clinical treatment outcomes. Additionally, clinical data were utilized to predict OS in AML-DN patients. To assess the prognostic impact, univariate logistic regression, Cox regression, and contribution coefficient analyses were performed, focusing on the expression ratio of the NKG2D^+^TIGIT^−^ Vδ1 T-cell subset and its relationship with clinical prognosis. AML, acute myeloid leukemia; TCGA, The Cancer Genome Atlas; PB, peripheral blood; AML-DN, *de novo* acute myeloid leukemia; CR, complete remission; HIs, healthy individuals; OS, overall survival.

Current cancer immunotherapies are primarily based on αβ T cells, which heavily rely on MHC-mediated presentation of tumor-associated peptides or unique neoantigens, thus limiting their effectiveness and applicability in various scenarios. After years of preliminary clinical research, γδ T cells are now being explored as a viable and promising approach for cancer immunotherapy (20). γδ T cells comprise a relatively small subset of T cells in the PB of adult individuals. While there is substantial interindividual variability, γδ T cells usually account for anywhere between 1% and 10% of CD3^+^ T cells in human blood (21). The present study provides a phenotypic analysis of the PB γδ population in patients with AML (**Figure 5**). Human γδ T cells can be divided into two main groups according to their TCR usage of the Vδ1 and Vδ2 chains. Our results are consistent with previous findings that Vδ2 accounts for the majority of γδ T cells in PB, whereas Vδ1 accounts for a smaller proportion. Apart from Vγ9/Vδ2 and Vδ1, cells expressing one of the remaining Vγ (Vγ2, 3, 4, 5, or 8) or Vδ (Vδ3, 4, 5, or 6) elements on their surface are extremely rare among peripheral blood gd T lymphocytes (22). Vδ3 cells represent a rare and poorly studied γδ T-cell subset in the blood that can expand in the liver (23). Sequencing of the Vδ complementarity-determining region 3 revealed that nearly all non-Vδ1/Vδ2 cells utilized Vδ3 and that the tumor-infiltrating γδ T-cell clonotypes were unique to each patient (24). While no statistically significant differences were detected across groups within the non-Vδ1/Vδ2 subpopulation, this subset displayed expression patterns closely mirroring those observed in both Vδ1 and Vδ2 T-cell subsets. Importantly, this work extends our previous findings by providing functional evidence for the immunoregulatory capacity of γδ T cells in AML pathogenesis. We found that TIGIT and Foxp3 were generally expressed higher in total γδ T cells from AML-DN and CR patients, while they had lower expressions in HIs. Compared with that in the HIs, the expression of NKG2D was decreased in γδ T cells including Vδ1 T cells and Vδ2 T cells from the AML-DN and CR patients. The expression patterns of NKG2D, TIGIT, and Foxp3 in the non-Vδ1/Vδ2 subset were highly similar to those observed in the total γδ T-cell population, as well as in the Vδ1 and Vδ2 subsets. Furthermore, we analyzed the expression of NKG2D, TIGIT, and Foxp3 and found that the pairwise combinations of these three immune checkpoints connected with OS in AML patients. Our results show that highly expressed NKG2D was associated with well OS. Among patients with low expression of NKG2D in the total γδ T-cell group, concomitant high expression of TIGIT correlated with poor OS. Patients with low expression of NKG2D in total γδ T cells or Vδ2 T cells, together with high expression of Foxp3, also related to poor OS. Notably, there was an increase in the frequencies of NKG2D^+^TIGIT^−^ Vδ1 cells and NKG2D^−^TIGIT^+^ Vδ2 cells in AML patients who achieved CR after chemotherapy, indicating the relevance of γδ T-cell subsets and clinical outcomes of AML patients. It provides new insights into the role of γδ T cells and immune checkpoint receptors, particularly TIGIT and NKG2D, in AML. Our findings highlight the complex dynamics of the immune microenvironment in AML and emphasize how immune checkpoint molecules, especially TIGIT, contribute to immune exhaustion, while NKG2D appears to support favorable clinical outcomes through its expression on γδ T cells.

γδ T cells are considered to have their niche at the crossroads of innate and adaptive immunity (25). They share features of the adaptive immune system, with their expression of clonally rearranged TCR genes, but at the same time are similar to innate immune cells, with the lack of need for antigen processing to activate their effector functions. Therefore, γδ T cells rapidly respond to TCR triggering. Moreover, γδ T cells frequently co-express functional receptors of innate immune cells, such as activating NK receptors such as NKG2D, NKp30, and/or NKp44, which directly trigger cytotoxic activity (26–29), in addition to certain Toll-like receptors (TLRs), which can provide costimulatory signals (30, 31). The NKG2D receptor also contributes to immune surveillance, as illustrated by increased tumor incidence in NKG2D-deficient mice (32). Therefore, different strategies were pursued to restore NKG2D-mediated recognition of malignant cells. In a recent study, anti-MICA and anti-MICB antibodies were used to inhibit the shedding of these ligands, resulting in enhanced NK cell cytotoxicity through NKG2D and additional FcγRIIIA activation (33). The loss or downregulation of NKG2D expression on immune cells is associated with poor prognosis and immune evasion, underscoring its importance in tumor surveillance (34, 35). As NKG2D is expressed on NK cells as well as on T-cell subsets, it may also represent a promising target for antibody-based immunotherapy (36).

TIGIT is another important immune checkpoint receptor that is expressed on effector CD4^+^ and CD8^+^ T cells, Tregs, and follicular T helper cells and is also found on NK and memory T cells. TIGIT binds to three ligands, including CD155 (PVR), CD112 (PVRL2, nectin-2), and CD113 (PVRL3), which are also parts of the PVR/NECTIN family (37, 38). In both mice and humans, TIGIT inhibits NK cell degranulation, cytokine production, and NK cell-mediated cytotoxicity against tumor cells expressing CD155 (12, 39). Some studies have shown that co-expression of TIGIT and PD-1 could lead to impaired protective anti-tumor responses; therefore, antibody co-blockade of TIGIT and PD-1 could enhance CD8^+^ T-cell effector function, resulting in significant tumor clearance (40, 41). In recent years, some studies have suggested that targeting immune checkpoints (ICs) can reverse the dysfunction of γδ T cells in the tumor microenvironment (TME) and enhance anti-tumor responses by improving γδ T-cell proliferation and activation, as well as boosting cytotoxicity (42). One of the emerging strategies for the treatment of TCM may be immunotherapy with Immune Checkpoint Blockades (ICBs) (43). Also recently, Hajiasghar-Sharbaf et al. showed that CD8^+^ T cells co-expressing PD-1 and TIGIT are highly frequent in chronic lymphocytic leukemia (CLL) (44). Another study showed an imbalance in the distribution of TIGIT and CD226 on γδ T cells, with a decrease in CD226^+^ γδ T cells and an increase in TIGIT^+^ γδ T cells in patients with AML-DN patients. In contrast, TIGIT^−^CD226^+^ γδ T cells were restored in AML patients who achieved CR after chemotherapy (45).

In this study, we further explored the distribution of TIGIT in different subpopulations of γδ T cells. Compared with the CR group, the tendency of the TIGIT^+^ Vδ1 subset expressing on the surface of the total γδ T-cell population was higher in the AML-DN group. It was also found that the tendency of TIGIT^+^ Vδ2 in γδ T cells was higher in the AML-DN group than in the CR group. Additionally, it was discovered that patients with AML-DN had a higher tendency of TIGIT^+^ Vδ2 in γδ T cells than did the CR group. This result may be supported by the findings of Brauneck et al. who found that γδ T cells in the bone marrow (BM) from patients with AML and MM showed an increased expression of the co-inhibitory molecules PD-1, TIGIT, TIM-3, or CD39 in contrast to HIs (16). Moreover, this study illustrated the increased expression of TIGIT on γδ T cells in AML-DN and CR patients, hypothesizing that these cells are functionally “exhausted”. These observations suggest that Vδ2 exhaustion may be a key driving factor in tumor immune evasion.

γδ T cells have different functional subsets, including regulatory T-cell subsets that express the Foxp3 (17). Foxp3-positive αβ T cells are traditional Tregs, and these cells have been observed to possess an immune regulatory function in patients (46, 47). The regulatory subset of γδ T cells that express Foxp3, termed γδ Tregs, has been reported to be at a low expression frequency in tumor-infiltrating leukocytes and human PB. However, the relevant underlying regulatory mechanism remains unclear. The number and functions of Tregs are reported to be enhanced in some solid tumors, facilitating immune escape (48, 49). It is reported that the percentages of Tregs were higher in AML-DN patients compared with HCs and AML patients in CR. Furthermore, AML-DN patients had higher mRNA expression of Foxp3 compared to that in AML patients in CR and HCs (19). Determining prognosis and treatment response in oncology patients drives personalized medicine strategies (50). Our previous study described the association between the expression levels of the *PD-1* and *FOXP3* genes and the OS in the BM leukemia cells AML patients based on TCGA database and described the expression pattern correlated with the poor OS (51). Consistently, our data also demonstrate an increasing trend in the Foxp3^+^ T-cell subsets in the Vδ1 and Vδ2 T-cell populations, which may be related to the primary reason for leukemia immunosuppression. Furthermore, our results show that high expression of NKG2D is associated with good OS, whereas high expression of TIGIT and Foxp3 is associated with poor survival. This finding has great importance for AML diagnosis and treatment. It also provides insight into how checkpoint dysregulation and subpopulation-specific depletion contribute to disease progression in other hematologic tumors or other malignancies.

In recent years, an increasing amount of basic research has focused on γδ T-cell development, antigen recognition, activation, and anti-tumor immune responses. In addition, the number of clinical trials of γδ T cell-based immunotherapy strategies is increasing (52). In addition to the previously reported that the high frequency of the PD-1^+^Foxp3^+^ γδ T subset is associated with poor clinical outcomes, we further speculate that the high frequency of the NKG2D^+^TIGIT^−^ Vδ1 T subset is associated with favorable clinical outcomes, which could reinforce evidence of a link between NKG2D and TIGIT in γδ T cells. These results support the idea that the blockade of inhibitory immune checkpoint receptors or the addition of a co-stimulatory signal through activation may improve AML patient survival. This study not only enhances our understanding of the mechanisms underlying γδ T-cell dysfunction within the immune microenvironment of AML but, more importantly, bridges basic research and clinical translation. By developing prognostic models based on the expression patterns of γδ T-cell checkpoints, designing immunomodulatory regimens tailored to specific subpopulations, and exploring cross-tumor combinatory therapeutic strategies, this work is poised to usher in a new era of immunotherapy for a range of malignant tumors, including AML. Although we found a correlation between γδ T-cell functional subpopulations and clinical outcomes, the sample size was insufficient and was not validated by functional assays. Further studies are planned in the future to incorporate *ex vivo* and *ex vivo* functional assays to directly assess the cytotoxic potential or immunosuppressive activity of these subpopulations and explore the efficacy of AML immunotherapy.

## Data Availability

The data presented in the study are deposited in the TCGA repository, accession number phs000178.

## Supplementary material

Supplementary Figure 1 Distribution and frequency of NKG2D, TIGIT, and Foxp3 expression in non-Vδ1/Vδ2 T-cell subset from PB of AML-DN patients, CR patients, and HIs. **(A)** Comparison of the percentages of γδ T cells and their subsets (Vδ1, Vδ2, non-Vδ1/Vδ2). **(B)** Distribution and frequency of NKG2D, TIGIT, and Foxp3 expression in non-Vδ1/Vδ2 subset. **(C)** Co-Expression of NKG2D and TIGIT in non-Vδ1/Vδ2 subset. **(D)** Co-Expression of NKG2D and Foxp3 in non-Vδ1/Vδ2 subset. Data are presented as medians. The data were analyzed using the unpaired Mann-Whitney U test **(A–D)**.

Supplementary Figure 2 Isotype control validation for NKG2D, TIGIT, and Foxp3 antibodies in γδ T cells by flow cytometry. Isotype-matched control staining for NKG2D, TIGIT, and Foxp3 antibodies in γδ T cells is shown for one AML-DN patient, one CR patient, and one HI. γδ T cells were gated from CD3^+^ T cells, and the specificity of the target proteins was validated by comparing with corresponding isotype controls. The samples are from the same donor cohorts as those shown in **Figure 3A**.
