# Supplementary material for: Heterogeneity of γδ T-cell subsets and their clinical correlation in patients with AML
Source: Front Immunol. 2025 Apr 1;16:1552235. doi: 10.3389/fimmu.2025.1552235 (PMC11996841; doi:10.3389/fimmu.2025.1552235)
Supplement: Supplementary Figure 1 — Distribution and frequency of NKG2D, TIGIT, and Foxp3 expression in non-Vδ1/Vδ2 T-cell subset from PB of AML-DN patients, CR patients, and HIs. (A) Comparison of the percentages of γδ T cells and their subsets (Vδ1, Vδ2, non-Vδ1/Vδ2). (B) Distribution and frequency of NKG2D, TIGIT, and Foxp3 expression in non-Vδ1/Vδ2 subset. (C) Co-Expression of NKG2D and TIGIT in non-Vδ1/Vδ2 subset. (D) Co-Expression of NKG2D and Foxp3 in non-Vδ1/Vδ2 subset. Data are presented as medians. The data were analyzed using the unpaired Mann-Whitney U test (A–D). [file DataSheet1.pdf]

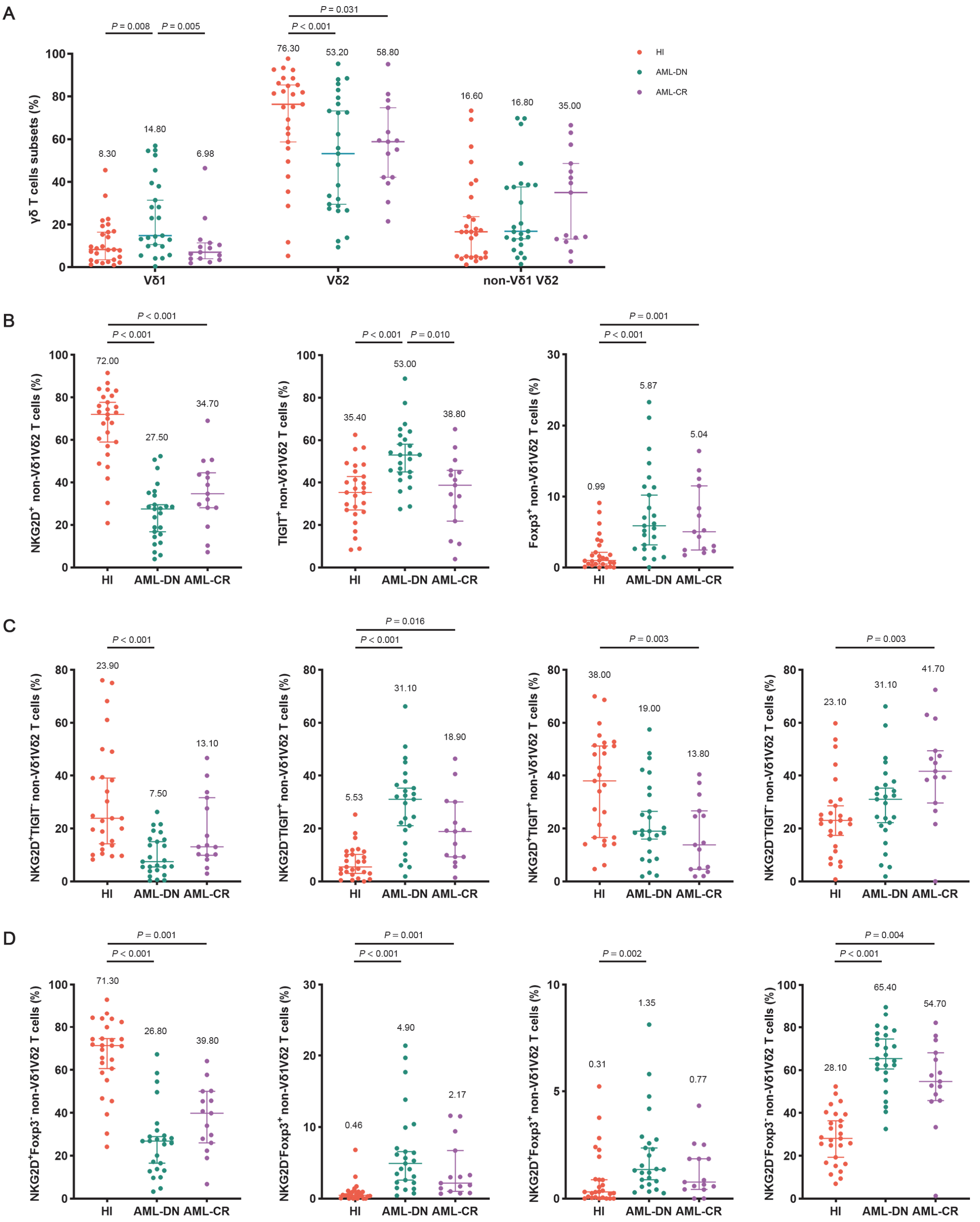

Supplementary Figure 1. Distribution and frequency of NKG2D, TIGIT, and Foxp3 expression in non-Vδ1/Vδ2 T-cell subset from PB of AML-DN patients, CR patients, and HIs. (A) Comparison of the percentages of γδ T cells and their subsets (Vδ1, Vδ2, non-Vδ1/Vδ2). (B) Distribution and frequency of NKG2D, TIGIT, and Foxp3 expression in non-Vδ1/Vδ2 subset. (C) Co-Expression of NKG2D and TIGIT in non-Vδ1/Vδ2 subset. (D) Co-Expression of NKG2D and Foxp3 in non-Vδ1/Vδ2 subset. Data are presented as medians. The data were analyzed using the unpaired Mann-Whitney U test (A to D).
