# Supplementary material for: Heterogeneity of γδ T-cell subsets and their clinical correlation in patients with AML
Source: Front Immunol. 2025 Apr 1;16:1552235. doi: 10.3389/fimmu.2025.1552235 (PMC11996841; doi:10.3389/fimmu.2025.1552235)
Supplement: Supplementary Figure 2 — Isotype control validation for NKG2D, TIGIT, and Foxp3 antibodies in γδ T cells by flow cytometry. Isotype-matched control staining for NKG2D, TIGIT, and Foxp3 antibodies in γδ T cells is shown for one AML-DN patient, one CR patient, and one HI. γδ T cells were gated from CD3+ T cells, and the specificity of the target proteins was validated by comparing with corresponding isotype controls. The samples are from the same donor cohorts as those shown in Figure 3A . [file DataSheet2.pdf]

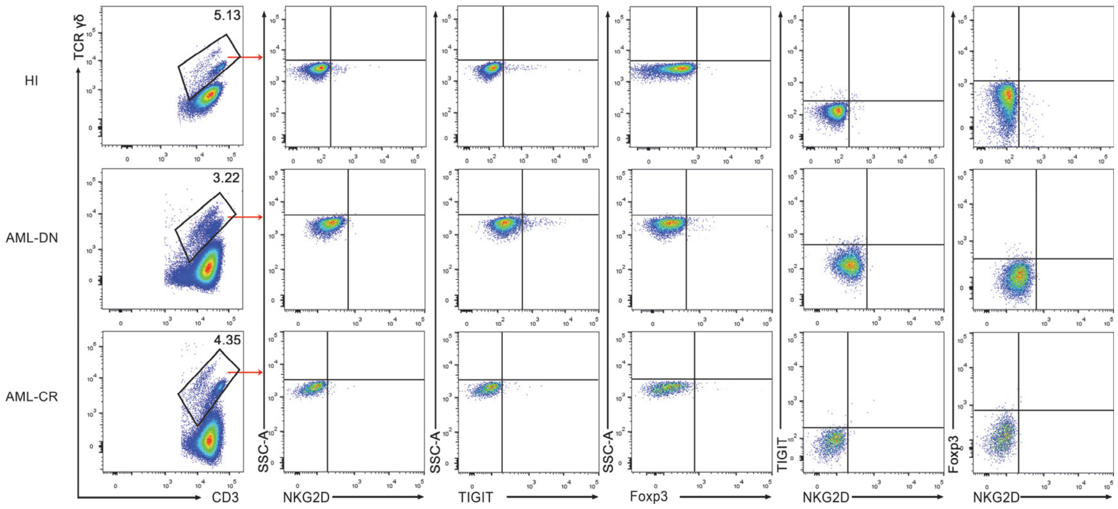

Supplementary Figure 2. Isotype control validation for NKG2D, TIGIT, and Foxp3 antibodies in γδ T cells by flow cytometry. Isotype-matched control staining for NKG2D, TIGIT, and Foxp3 antibodies in γδ T cells is shown for one AML-DN patient, one CR patient, and one HI. γδ T cells were gated from $CD3^+$ T cells, and the specificity of the target proteins was validated by comparing with corresponding isotype controls. The samples are from the same donor cohorts as those shown in Figure 3A.
